# One-Step Preparation of Adhesive Composite Hydrogels through Fast and Simultaneous In Situ Formation of Silver Nanoparticles and Crosslinking

**DOI:** 10.3390/gels8050256

**Published:** 2022-04-21

**Authors:** Yi Li, Yunchao Xiao, Man Xi, Guibin Li, Yang Jiang

**Affiliations:** 1 College of Materials and Textile Engineering & Nanotechnology Research Institute (NRI), Jiaxing University, Jiaxing 314001, China; liyi@zjxu.edu.cn (Y.L.); yunchao.xiao@zjxu.edu.cn (Y.X.); xixi1228@163.com (M.X.); 2 Key Laboratory of Medical Electronics and Digital Health of Zhejiang Province, Engineering Research Center of Intelligent Human Health Situation Awareness of Zhejiang Province, Jiaxing University, Jiaxing 314001, China

**Keywords:** composite hydrogels, silver nanoparticles, adhesive, one-step preparation

## Abstract

In this study, a series of gelatin/silver nanoparticles (AgNPs) composite hydrogels are prepared for the first time through the facile in situ formation of AgNPs. AgNPs, which are formed by reducing Ag^+^ using dopamine-conjugated gelatins. These can simultaneously crosslink gelatin molecules, thus generating three-dimentional and porous hydrogels. The gelation time and pore sizes of these composite hydrogels can be controlled by controlling the feeding concentration of AgNO_3_ and weight content of gelatin in water, respectively. The feeding concentration of AgNO_3_ also has an effect on the equilibrium swelling ratio of the hydrogels. Moreover, these composite hydrogels, with a controllable gelation time and in situ forming ability, exhibit good adhesive properties and can be used as drug-release depots.

## 1. Introduction

Hydrogels are soft materials with a porous network structure and ability to absorb a large amount of aqueous fluid [1,2,3,4]. Due to their unique properties, hydrogels have been widely used in a number of applications including drug delivery, tissue engineering, and regenerative medicine. Based on their crosslinking mechanisms, hydrogels can be classified into physically crosslinked and chemically crosslinked hydrogels [5,6,7,8,9]. Physically crosslinked hydrogels are generally prepared through physical interactions, such as hydrophobic interactions, static electronic interactions, hydrogen bondings, etc. The use of small-molecule crosslinkers can be avoided in the fabrication of this kind of hydrogel, but their mechanical properties are generally poor. Chemically crosslinked hydrogels are prepared the through formation of chemical bonds, and the mechanical properties of this kind of hydrogels are stronger. Current hydrogel materials still have a number of problems that limit their real applications, so it is important to explore new fabrication methods and new hydrogel properties.

Due to some unique properties of silver nanoparticles (AgNPs) (such as their antibacterial activity and conductivity), AgNPs-incorporated hydrogels have been widely studied and applied in drug delivery, wound healing, water purification, and tissue engineering [10,11,12,13,14,15,16,17,18]. The most common method used to fabricate AgNPs-incorporated hydrogels involves crosslinking pre-formed AgNPs in aqueous solutions. For example, Ren and coworkers prepared a AgNPs-incorporated hydrogel by crosslinking polymer-coated AgNPs with α-cyclodextrins [19]. This hydrogel showed good thermal responsibility, injectability, antibacterial activity, and biocompatibility. Gabilondo and coworkers developed a biocompatible hydrogel with covalently embedded AgNPs through chemical reactions between gelatin and functionalized AgNPs [20]. Their hydrogel had a high storage modulus and low swelling ratio and could be used as a drug delivery system. Another method that can be used to fabricate AgNPs-incorporated hydrogels involves physically entrapping AgNPs in pre-formed hydrogel networks. For example, Fan et al. reported an AgNPs-incorporated hydrogel by reducing Ag^+^ with trisodium citrate in a pre-formed chitosan hydrogel [21]. Their hydrogel had high mechanical and antibacterial properties and could potentially be used for wound healing. Chen et al. fabricated a poly(acrylic acid)/AgNPs composite hydrogel by reducing Ag^+^ with NaOH in a pre-formed poly(acrylic acid) hydrogel [22]. This composite hydrogel could completely inhibit bacteria and yeast proliferation, meaning that it has potential in the development of superabsorbent antimicrobial pharmaceutical products, as well as in the treatment of infected wounds.

In this work, we report a novel preparation method for gelatin/AgNPs composite hydrogels through the in situ formation of AgNPs. AgNPs that are formed by reducing Ag^+^ using dopamine-conjugated gelatin can simultaneously crosslink gelatin molecules, thus generating hydrogels. The gelation time and pore sizes of these composite hydrogels can be controlled by controlling the feeding concentration of AgNO_3_ and weight content of gelatin in water, respectively. The feeding concentration of AgNO_3_ also has an effect on the equilibrium swelling ratio of the hydrogels. Moreover, these composite hydrogels exhibit good adhesive properties and can be used as drug-delivery depots. The method reported in this work is eco-friendly (no organic solvent is needed in the whole process) and has advantages in its simplification of fabrication process of AgNPs-incorporated hydrogels, as well as controlling the micro-structures and properties of hydrogels. However, since vigorous stirring is required for the uniform distribution of AgNPs in the hydrogel matrix, it is difficult to control the shapes of the resulting hydrogels.

## 2. Results and Discussion

### 2.1. Synthesis of DA-GLTs

Gelatin is a mixture of polypeptides derived from collagen. Due to the presence of glutamic acid units, gelatin contains numerous carboxyl groups on its backbone. Here, dopamine was conjugated onto gelatin through chemical reactions between the carboxyl groups of gelatin and the primary amine groups of dopamine, using EDC/NHS as a coupling agent. To investigate the influence of dopamine conjugation density on the properties of composite hydrogels, the feeding weight ratio of gelatin to dopamine was controlled (Table S1), and the feeding molar ratio of EDC:NHS:DA was fixed at 1:1:1.3. Figure S1 shows ^1^H NMR spectra of DA-GLTs with different dopamine contents (DA-GLT_0.5_, DA-GLT_0.75_, and DA-GLT_1_). This figure indicates that peaks corresponding to the protons on the benzyl rings of dopamine clearly appear between 7.0 and 7.25 ppm, which confirms the successful conjugation of dopamine onto gelatin. Furthermore, the peak intensity ratio of gelatin to DA decreases in the order of DA-GLT_0.5_, DA-GLT_0.75_, and DA-GLT_1_. For example, the peak intensity ratios of the peaks near 0.75 ppm (corresponding to the protons of gelatin) to the peaks near 7.2 ppm (corresponding to the protons of DA) are 5.9:1, 4.6:1, and 3.1:1 for DA-GLT_0.5_, DA-GLT_0.75_, and DA-GLT_1_, respectively. This confirms that the conjugation density of DA can be tuned by controlling the feed ratio of gelatin to DA.

### 2.2. Preparation of AgNPs-Crosslinked Composite Hydrogels

**Scheme 1:**
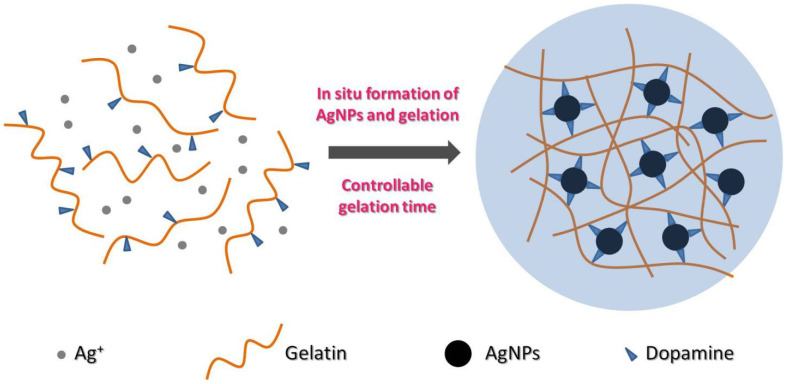
One-step and facile preparation of adhesive gelatin/AgNPs composite hydrogels.

AgNPs-crosslinked gelatin hydrogels are prepared in a simple one-step procedure through fast and simultaneous AgNPs formation and crosslinking. A previous study reported that dopamine can reduce Ag^+^ in aqueous solutions, leading to the formation of AgNPs [19]. It is believed that the hydroxyl groups of dopamine can rapidly reduce Ag^+^ in aqueous solutions. In this procedure, the dihydroxy molecules of dopamine lose two electrons, leading to the generation of a quinone molecule, and the quinone molecules are subsequently adsorbed on the surface of the formed AgNPs. For the formation of AgNPs in this work, we used gelatin with pendent dopamine groups (Scheme 1). Since there are many pendent dopamine groups in one molecular chain of gelatin, gelatin can connect different AgNPs. We can control the gelation and gelation time by controlling the concentration of DA-GLT, conjugation density of dopamine, and weight ratio of DA-GLT to AgNO_3_.

As shown in Table 1, we fixed the weight ratio of DA-GLT to AgNO_3_ at 50:11 and changed the concentration of DA-GLT and conjugation density of DA. This table shows that, when the concentration of DA-GLT is 5 wt%, no gelation is observed at any DA conjugation density. Similarly, when DA-GLT_0.1_ is used, no gelation is observed, even at a DA-GLT_0.1_ concentration of 20 wt%. Gelation is more likely to occur at higher DA-GLT concentrations and higher conjugation densities of DA. Furthermore, we can also see from Table 1 that the gelation time (ranging from several seconds to several minutes) can be controlled by controlling the concentration of DA-GLT and the conjugation density of DA. Specifically, a higher DA-GLT concentration and higher conjugation density of DA led to a faster gelation. This controllable gelation time is crucial for the in situ formation of injectable hydrogels, since too fast a gelation leads to a needle clogging problem, while too slow a gelation causes the spread of the solution to undesired sites [23,24].

**Figure 1:**
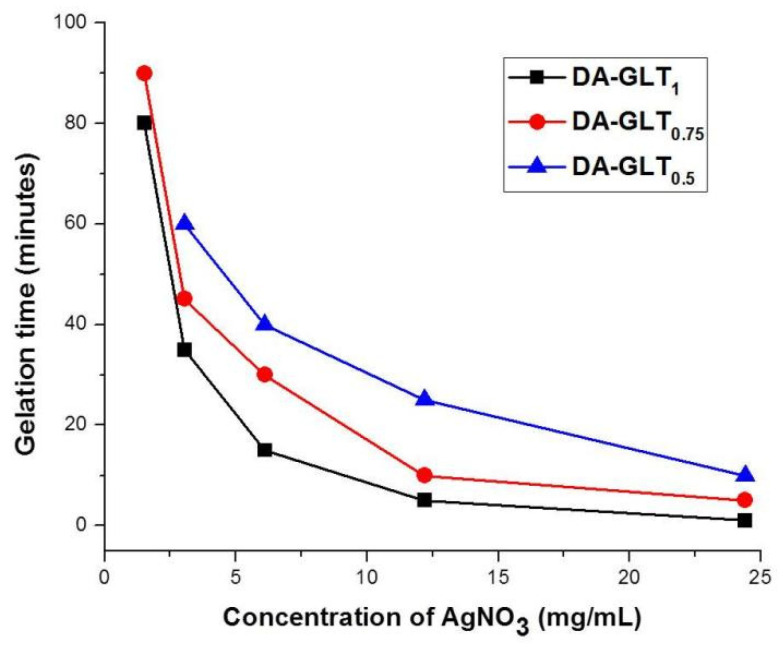
Relationships between gelation time and the feeding concentrations of AgNO_3_ at different DA conjugation densities.

In order to further explore whether gelation can occur at low AgNO_3_ concentrations, we fixed the concentration of DA-GLT at 10 wt% and changed the concentration of AgNO_3_ and conjugation density of DA (Table S2). It can be clearly seen that the gelation time increases with decreasing concentrations of AgNO_3_, which might be attributed to the formation of fewer AgNPs as crosslinking centers. However, gelation occurs even at a AgNO_3_ concentration of as low as 1.525 mg/mL, although the gelation time is substantially longer. In addition, at the same feeding concentration of AgNO_3_, the gelation time decreases with the increasing conjugation density of DA. Figure 1 illustrates the relationships between gelation time and the feeding concentration of AgNO_3_ at different DA conjugation densities. The gelation time decreases quickly when the feeding concentration of AgNO_3_ increases in the beginning. However, further increases in the feeding concentration of AgNO_3_ do not decrease the gelation time as much.

### 2.3. Surface Morphologies and Thermogravimetric Analysis (TGA) of Freeze-Dried Composite Hydrogels

**Figure 2:**
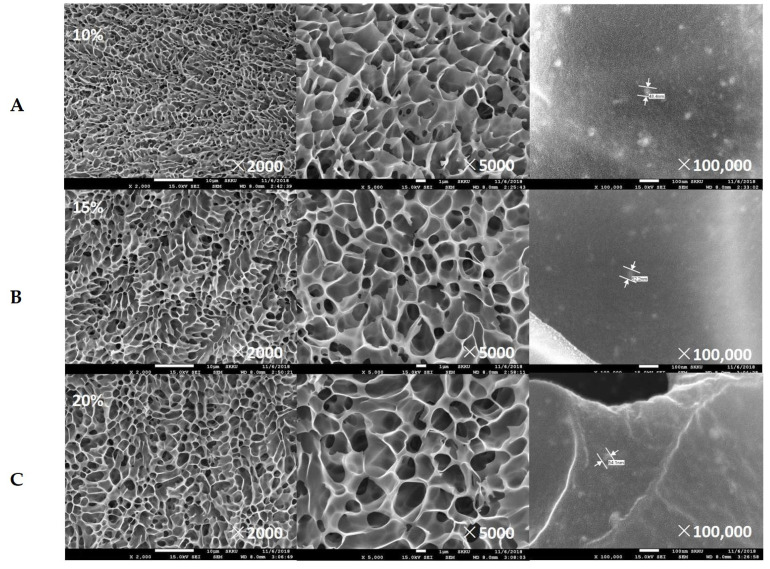
SEM images of freeze-dried DA-GLT_1_/AgNPs composite hydrogels with different weight contents of DA-GLT_1_ ((**A**): 10 wt%; (**B**): 15 wt%; and (**C**): 20 wt%) and different magnifications (2000, 5000, and 100,000).

The surface morphologies of the freeze-dried gelatin/AgNPs composite hydrogels with different contents of DA-GLT_1_ (10 wt%, 15 wt%, and 20 wt%) were observed using FE-SEM with a feeding weight ratio of DA-GLT_1_:AgNO_3_ fixed at 50:11. As shown in Figure 2, all three composite hydrogels exhibited a porous structure, indicating the successful formation of uniform gelatin networks in the composite hydrogels. Interestingly, the pore sizes of the hydrogels increased with increasing DA-GLT_1_ contents, which might be attributed to the formation of more crosslinking centers, which makes the supporting solid parts aggregate more tightly. At a magnification time of 100,000, we can see the presence of AgNPs with a spherical shape and uniform distribution inside the gelatin matrix. The sizes of the AgNPs in all three saples were about 50 nm, which can be attributed to the fact that the feeding ratios of DA:AgNO_3_ were the same. Elemental analysis based on EDS confirmed the presence of the Ag element, and the weight content of Ag atoms was about 16.8 wt% in the freeze-dried composite hydrogels.

The weight percentage of AgNPs in the composite hydrogel was further determined by TGA. As shown in Figure S2, the remaining weight percentage of the composite hydrogel residues at 1000 °C is about 34.5 wt%. Since the residues contain both AgNPs and NO_3_^−^, the weight percentage of AgNPs is calculated to be about 21.9 wt%. This result is similar to the value obtained from EDS.

### 2.4. Viscosities and Swelling Behaviors of Composite Hydrogels

**Figure 3:**
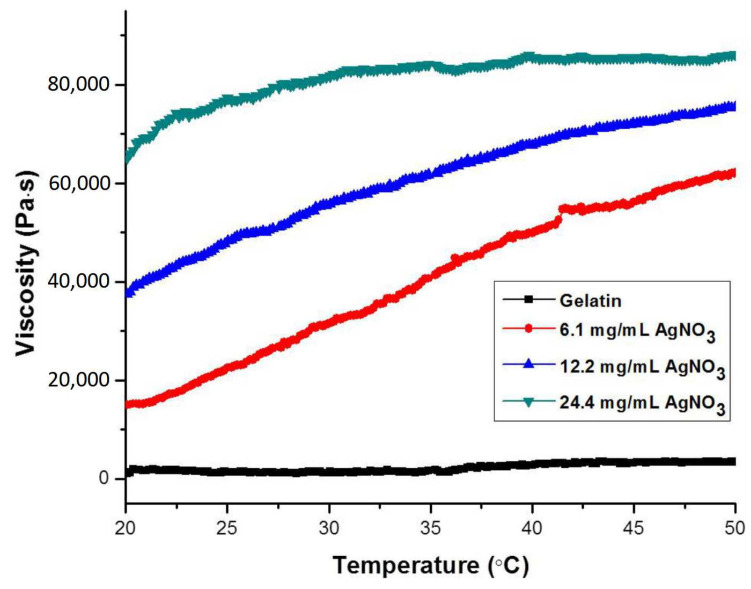
Temperature-dependent viscosities of gelatin solution and DA-GLT_1_/AgNO_3_ composite hydrogels at different feeding concentrations of AgNO_3_.

The mechanical properties of the composite hydrogels were studied by measuring the temperature-dependent viscosities of the composite hydrogels with different feeding concentrations of AgNO_3_. As shown in Figure 3, the viscosity of the gelatin solution remained low at any temperature ranging from 20 °C to 50 °C. Following the addition of different amounts of AgNO_3_ solutions (6.1 mg/mL, 12.2 mg/mL, and 24.4 mg/mL), the viscosities increased dramatically due to the formation of hydrogels. In addition, the viscosity increases with increasing feeding concentrations of AgNO_3_, which can be attributed to the increased crosslinking density caused by the formation of more AgNPs. Interestingly, the viscosities of all three composite hydrogels were shown to gradually increase with increasing temperatures. This might be explained by the stronger interactions between the formed quinone and AgNPs at higher temperatures. At body temperature (37 °C), these materials have high viscosities that are suitable for use in situ, forming injectable hydrogels in various biomedical applications.

**Figure 4:**
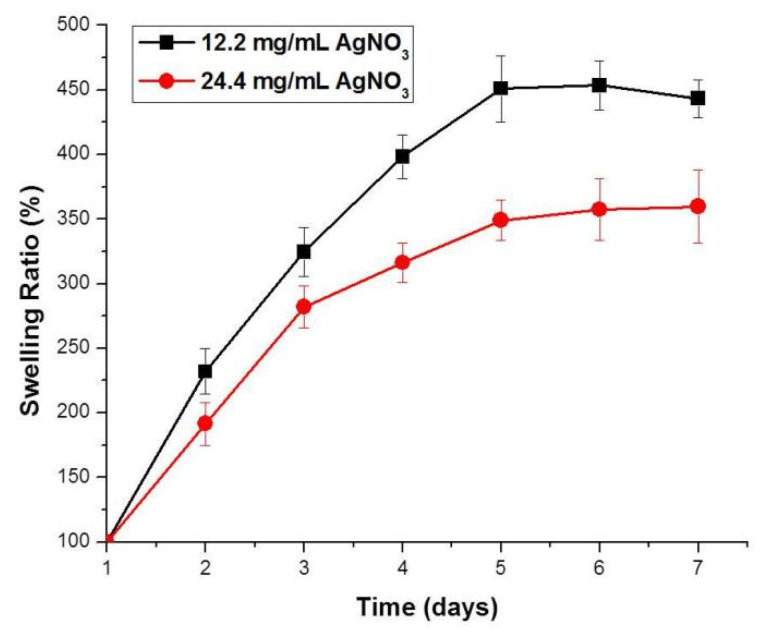
Time-dependent swelling ratios of DA-GLT_1_/AgNPs hydrogels with different feeding concentrations of AgNO_3_.

The swelling behaviors of DA-GLT_1_/AgNPs hydrogels with two different feeding concentrations of AgNO_3_ were studied. As shown in Figure 4, for both composite hydrogels, the swelling ratios gradually increase with time, and equilibrium swellings are reached after about four days. It should be noted that the equilibrium swelling ratio of the composite hydrogel with a higher feeding concentration of AgNO_3_ (about 350%) is lower than that of the composite hydrogel with a lower feeding concentration of AgNO_3_ (about 450%), which is attributed to the more rigid network caused by a higher crosslinking density. Swelling ratio is a very important hydrogel parameter, and different swelling ratios are required for different application purposes [25].

### 2.5. Adhesive Properties of Composite Hydrogels

**Figure 5:**
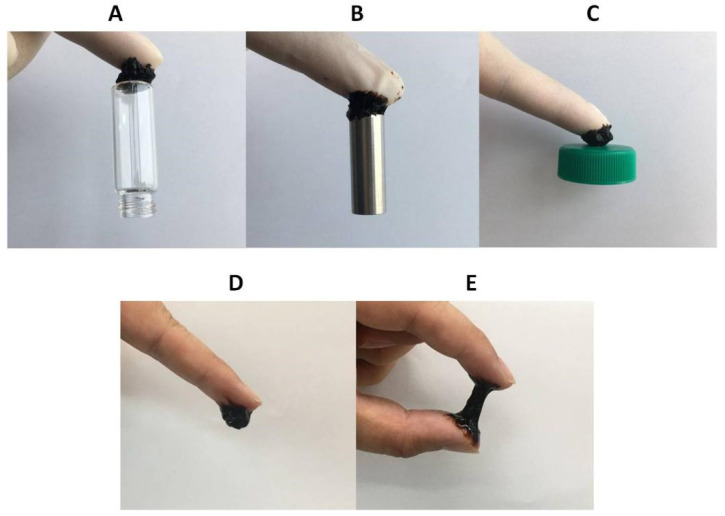
Adhesion of DA-GLT/AgNPs composite hydrogels to (**A**) glass, (**B**) metal, (**C**) plastic, and (**D**,**E**) skin.

The adhesive property is an important property for hydrogels that are used as binders, drug-delivery carriers, wound dressings, etc. In this work, the adhesive property of the DA-GLT/AgNPs composite hydrogels was tested on various solid substrates. As shown in the photos in Figure 5, the composite hydrogels can adhere to many different common materials with different chemical and physical properties, such as rubber, glass, metal, plastic, and skin. Since gelatin hydrogels are not adhesive, the versatile adhesive properties of the composite hydrogels might be attributed to the presence of dopamine groups and the formation of hydrogen bonds between the hydrogels and the substrates. As can be seen from Figure 5E, this DA-GLT/AgNPs composite hydrogel is stretchable. The adhesion between the hydrogel and skin is even stronger than the strength of the hydrogel, which means that stretching the hydrogel further results in a break in the middle of the hydrogel, instead of detachment from the skin.

### 2.6. Sustained Release of Model Drugs from Composite Hydrogels

**Figure 6:**
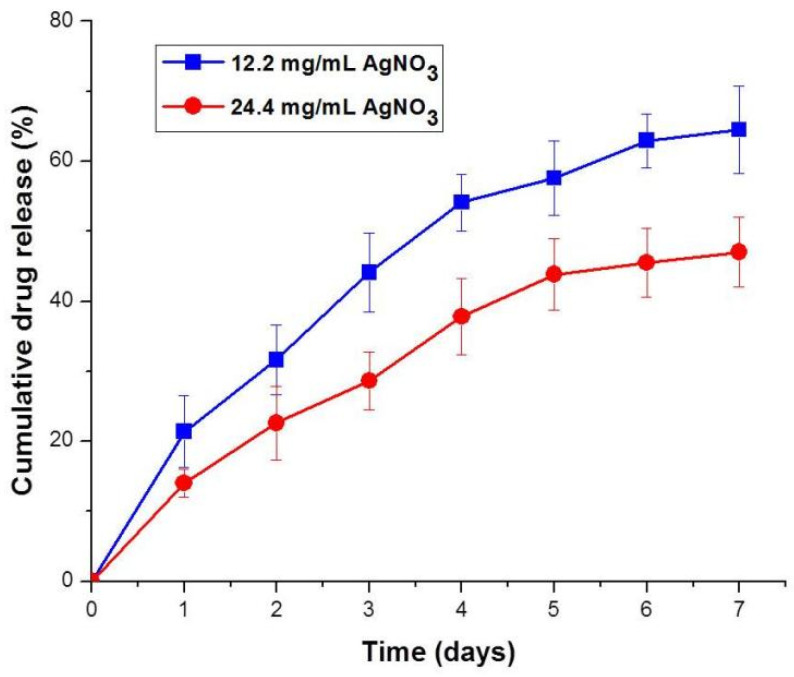
Cumulative drug release from DA-GLT_1_/AgNPs composite hydrogels with different feeding concentrations of AgNO_3_.

The composite hydrogels were tested as depots for the sustained release of therapeutic agents. Figure 6 shows the time-dependent cumulative release of DOX from the composite hydrogels with different feeding concentrations of AgNO_3_. DOX (in its salt form) is a widely used, water-soluble drug in cancer treatments. As shown in Figure 6, DOX can be released from both composite hydrogels in a sustained manner without any initial burst release. This might be attributed to the fact that electrostatic interactions between anionic gelatin and cationic DOX restrict free movement of the DOX molecules. Furthermore, the release rate of DOX from the composite hydrogel with higher feeding concentration of AgNO_3_ is slightly slower than that from the composite hydrogel with a lower feeding concentration of AgNO_3_, which might be attributable to the lower swelling ratio of the composite hydrogel with a higher feeding concentration of AgNO_3_. This result suggests that these composite hydrogels are good candidates for controlled drug release depots.

## 3. Conclusions

A series of gelatin/AgNPs composite hydrogels are prepared in one-step procedures through fast and simultaneous AgNPs formation and crosslinking. AgNPs formed by the in situ reduction of Ag^+^ with DA are embedded in the gelatin matric, which is a driving force of the formation of chemically crosslinked composite hydrogels. The weight content of DA-GLTs, concentration of AgNO_3_, and conjugation density of DA are tuned to investigate the gelation behaviors (gelation time and pore sizes), as well as the properties of the formed composite hydrogels (viscosities and swelling behaviors). Due to the controllable gelation time, these hydrogels can be used for the in situ formation of injectable hydrogels. These composite hydrogels can adhere to different types of substrates, such as rubber, glass, metal, and plastic. Moreover, these composite hydrogels have great potential for use as injectable depots for sustained drug release.

## 4. Experimental

### 4.1. Materials

Gelatin (from cold-water fish skin), dopamine hydrochloride (DA·HCl), N-hydroxysuccinimide (NHS, 98%), and silver nitrate (AgNO_3_, 99%) were obtained from Sigma-Aldrich (St. Louis, MO, USA). 1-(3-Dimethylaminopropyl)-3-ethylcarbodiimide hydrochloride (EDC·HCl, 98%) was purchased from Tokyo Chemical Industries (Tokyo, Japan). All of the chemicals were used without further purification.

### 4.2. Synthesis of Dopamine-Conjugated Gelatin (DA-GLT)

First, 3 g gelatin and 0.5 g DA·HCl (0.0026 mol) were dissolved in 100 mL of deionized water (DIW). Next, 0.375 g EDC·HCl (0.002 mol) and 0.225g NHS (0.002 mol) were added into the above solution under vigorous stirring. The reaction was then allowed to stand at room temperature in a N_2_ atmosphere. Twenty-four hours later, the solution, containing a mixture of reagents and products, was transferred to a dialysis bag (MWCO 3500 Da). After dialysis against DIW for 24 h, the DA-GLT was obtained by lyophilization. DA-GLTs with different dopamine contents were synthesized in the same manner by proportionally changing the feeding amounts of EDC·HCl, NHS, and DA·HCl.

### 4.3. Preparation of Gelatin/AgNPs Hydrogels

A series of gelatin/AgNPs hydrogels was prepared by tuning the concentration of gelatin and the ratio of gelatin to AgNPs. First, 0.1 g DA-GLT was dissolved into 0.79 mL DIW in a vial. Next, 0.11 mL AgNO_3_ solution with AgNO_3_ concentration of 0.2 mg/mL was quickly added into the gelatin solution under vortexing (the final mass concentration of gelatin in DIW was 10 wt%). A hydrogel was considered to be formed if the gelatin solution was not flowable within one minute when the vial was inverted.

### 4.4. Characterizations of DA-GLTs and Composite Hydrogels

#### 4.4.1. Characterization of DA-GLTs

First, 0.01 g DA-GLT was dissolved in 0.99 g deuterium oxide (D_2_O, 1 wt%). The successful conjugation of dopamine onto gelatin and the contents of dopamine were determined by proton nuclear magnetic resonance spectroscopy (^1^H NMR) measurement on a Varian Unity Inova 500 instrument (500 MHz, Varian, Palo Alto, CA, USA).

#### 4.4.2. Surface and Elemental Analysis

The surface morphologies of the freeze-dried composite hydrogels were observed using a high-resolution field emission scanning electron microscope (HR FE-SEM; JSM-7600F, JEOL, Tokyo, Japan). Next, samples were sputtered with Au (sputtering tine was 60 s). Then, the content of Ag was analyzed using energy-dispersive spectroscopy (EDS; JSM-7600F, JEOL, Tokyo, Japan).

#### 4.4.3. Thermogravimetric Analysis (TGA)

TGA of freeze-dried composite hydrogels was conducted using a Seiko Exstar 6000 (TG/DTA 6100, Seiko, Tokyo, Japan) under N_2_ purge from room temperature to 1000 °C at a heating rate of 10 °C/min.

#### 4.4.4. Dynamic Rheological Measurement of Hydrogels

The mechanical properties of the composite hydrogels at different temperatures (raised at a rate of 0.2 °C/min) were assessed by measuring the viscosity change with a dynamic mechanical analyzer (Bohlin Rotational Rheometer, Malvern Panalytical Ltd., Malvern, United Kingdom) in oscillation mode. In brief, composite hydrogels were placed between an upper plate (20 mm in diameter) and a bottom plate (100 mm in diameter) with a gap of 0.25 mm. Then, a controlled shear stress of 0.4 Pa was applied at an oscillation frequency of 1 rad/s.

#### 4.4.5. Swelling Behaviors of Composite Hydrogels

First, a piece of pre-weighed gelatin/AgNPs composite hydrogel was immersed in DIW. Next, at different timepoints, the swollen hydrogel was taken out and its weight was measured using an analytical balance. Then, the swollen hydrogel was immersed in fresh DIW again. The swelling ratio (SR) of the hydrogels was calculated using the equation: SR (%) = (W_s_−W_0_)/W_0_ × 100%, where W_s_ and W_0_ are the weights of the swollen and initial hydrogels, respectively. Equilibrium swelling was considered to be reached when the SR value did not increase further.

#### 4.4.6. Adhesive Properties of Composite Hydrogels

The composite hydrogels were placed on different substrates such as rubber, metal, glass, skin, and plastic. The adhesion of the composite hydrogels on each substrate was visually observed.

#### 4.4.7. Drug Release Behaviors

In order to test the ability of gelatin/AgNPs composite hydrogels as drug release depots, doxorubicin hydrochloride (DOX, an anticancer drug) was used as a model drug. To prepare drug-loaded hydrogels, 0.1 g DA-GLT, together with DOX, was dissolved into 0.79 mL phosphate buffer saline (PBS) in a vial. Next, 0.11 mL AgNO_3_ solution with AgNO_3_ concentration of 0.1 mg/mL or 0.2 mg/mL was quickly added into the gelatin solution under vortexing. Following the formation of hydrogels, 2 mL PBS solution was added to each vial, and the vial was then incubated in a water bath at 37 °C. At predetermined timepoints, the PBS solution containing released DOX was taken out and replaced with 2 mL of PBS solution. The concentration of the released DOX was analyzed using UV-visible spectroscopy at an excitation wavelength of 495 nm.

## Figures and Tables

**Table 1:** Gelation time of different DA-GLT at different contents of DA-GLT (the weight ratio of DA-GLT to AgNO_3_ in feed is fixed at 50:11).

	5 wt% (DA-GLT)	10 wt% (DA-GLT)	15 wt% (DA-GLT)	20 wt% (DA-GLT)
**DA-GLT_0.1_**	No gelation	No gelation	No gelation	No gelation
**DA-GLT_0.25_**	No gelation	No gelation	10 min	5 min
**DA-GLT_0.5_**	No gelation	10 min	1 min	<5 s
**DA-GLT_0.75_**	No gelation	5 min	<5 s	<5 s
**DA-GLT_1_**	No gelation	1 min	<5 s	<5 s

## Supplementary Materials

The following supporting information can be downloaded at: https://www.mdpi.com/article/10.3390/gels8050256/s1, Table S1: Feeding amounts of gelatin and DA·HCl in the synthesis of different DA-GLTs; Figure S1: ^1^H NMR spectra of DA-GLTs with different conjugation densities of DA.; Table S2: Gelation time of different DA-GLTs at different feeding concentrations of AgNO_3_; Figure S2: TGA curve of freeze-dried DA-GLT_1_/AgNPs composite hydrogel (10 wt% and a feeding AgNO_3_ concentration of 24.4 mg/mL).
