# Supplementary material for: One-Step Preparation of Adhesive Composite Hydrogels through Fast and Simultaneous In Situ Formation of Silver Nanoparticles and Crosslinking
Source: Gels. 2022 Apr 21;8(5):256. doi: 10.3390/gels8050256 (PMC9141602; doi:10.3390/gels8050256)
Supplement: Supplementary file 1 [file gels-08-00256-s001.zip › gels-1657733-supplementary.pdf]

gels

Article

One-Step Preparation of Adhesive Composite Hydrogels through Fast and Simultaneous In Situ Formation of Silver Nanoparticles and Crosslinking

Yi Li [1], Yunchao Xiao [1], Man Xi [1], Guibin Li [2,]* and Yang Jiang [1,]*

[1] College of Materials and Textile Engineering & Nanotechnology Research Institute (NRI), Jiaxing University, Jiaxing 314001, Zhejiang Province, PR China; liyi@zjxu.edu.cn (Y.L.); yunchao.xiao@zjxu.edu.cn (Y.X.); xixi1228@163.com (M.X.)

[2] Key Laboratory of Medical Electronics and Digital Health of Zhejiang Province and Engineering Research Center of Intelligent Human Health Situation Awareness of Zhejiang Province, Jiaxing University, Jiaxing 314001, Zhejiang Province, PR China

* Correspondence: 00008007@zjxu.edu.cn (G.L); jiangyang1981@126.com (Y.J.)

Supporting Information

Table S1. Feeding amounts of gelatin and DA·HCl in the synthesis of different DA-GLTs.

	Gelatin (g)	EDC·HCl (g)	NHS (g)	DA·HCl (g)	Molar ratio of EDC, NHS and DA in feed
**DA-GLT$_{0.1}$**	3	0.0375	0.0225	0.05	1:1:1.3
**DA-GLT$_{0.25}$**	3	0.09375	0.05625	0.125	1:1:1.3
**DA-GLT$_{0.5}$**	3	0.1875	0.1125	0.25	1:1:1.3
**DA-GLT$_{0.75}$**	3	0.28125	0.16875	0.375	1:1:1.3
**DA-GLT$_{1}$**	3	0.375	0.225	0.5	1:1:1.3

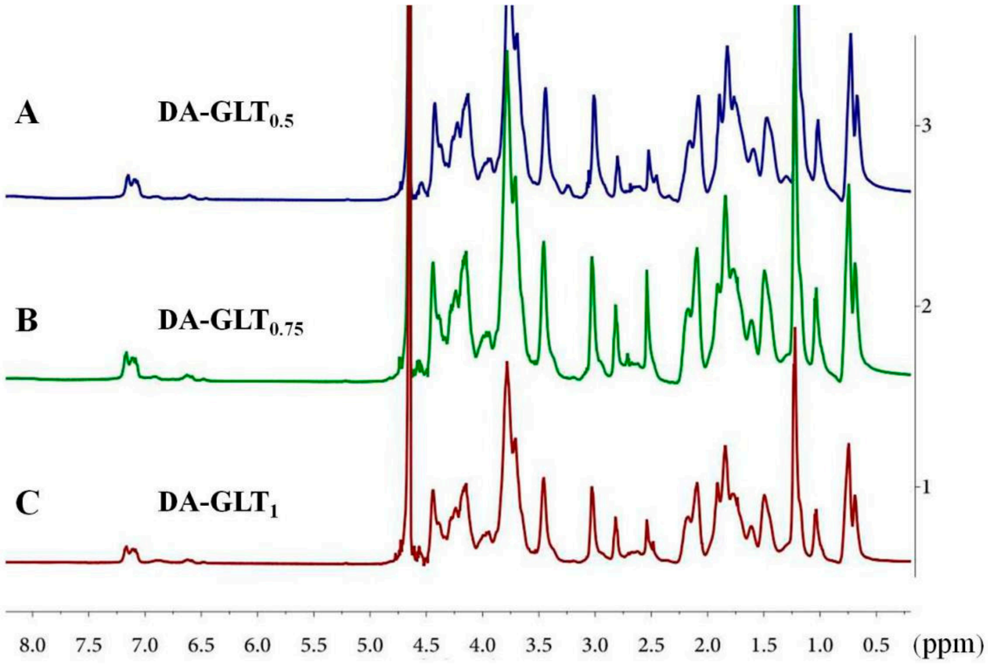

Figure S1. ^{1}H NMR spectra of DA-GLTs with different conjugation densities of DA.

Table S2. Gelation time of different DA-GLTs at different feeding concentrations of $AgNO_3$.

	**1.525 mg/mL $AgNO_3$**	**3.05 mg/mL $AgNO_3$**	**6.1 mg/mL $AgNO_3$**	**12.2 mg/mL $AgNO_3$**	**24.4 mg/mL $AgNO_3$**
**DA-GLT$_{0.1}$**	No gelation	No gelation	No gelation	No gelation	No gelation
**DA-GLT$_{0.25}$**	No gelation	No gelation	No gelation	No gelation	No gelation
**DA-GLT$_{0.5}$**	No gelation	60 min	40 min	25 min	10 min
**DA-GLT$_{0.75}$**	90 min	45 min	30 min	10 min	5 min
**DA-GLT$_{1}$**	80 min	35 min	15 min	5 min	1 min

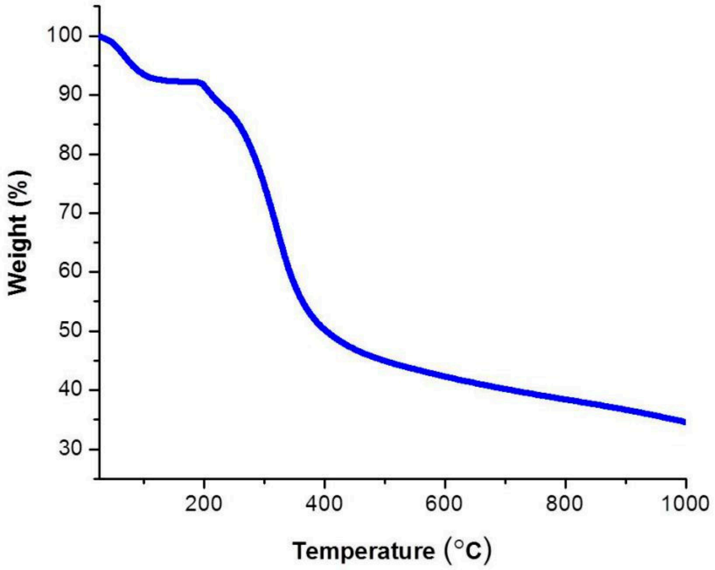

Figure S2. TGA curve of freeze-dried DA-GLT$_1$/AgNPs composite hydrogel (10 wt% and a feeding $AgNO_3$ concentration of 24.4 mg/mL).
